# Training in the Era of EHR: Examining the Experience of Medical Student Documentation in the Ambulatory Care Setting

**DOI:** 10.15694/mep.2020.000056.1

**Published:** 2020-03-27

**Authors:** Charlton Tsai, Julia Bellantoni, Omar Martinez-Uribe, Bruce Peyser

**Affiliations:** 1 Duke University School of Medicine

**Keywords:** medical student, documentation, electronic health record

## Abstract

This article was migrated. The article was marked as recommended.

**Purpose:** Documenting clinical encounters in the electronic health record has become an important component of medical student training. Reflecting this trend, recent rule changes by the Centers for Medicare and Medicaid services now permit billing for medical student notes. We sought to investigate the educational value of student note-writing following implementation of these changes.

**Methods:** We surveyed medical students at a private research university who participated in longitudinal ambulatory care experiences. Survey questions assessed the incorporation of student note-writing into clinic workflow, as well as the benefits and disadvantages of note-writing.

**Results:** Thirty-six students completed the survey. A majority of students perceived benefits in regards to residency preparedness, engagement with the clinical team, and clinical reasoning ability as a result of writing notes in clinic. While some students reported seeing fewer patients as a result of note-writing, most felt that use of the electronic health record did not negatively impact patient interaction. Barriers cited included a lack of knowledge regarding billing requirements and preceptor apprehension toward student note-writing.

**Conclusion:** The results of this study indicate that student note-writing continues to be a valuable part of medical training following recent billing changes. Our results also identify areas for improvement, including clarifying billing requirements and assuaging preceptor concerns.

## Introduction

Documentation in the electronic health record (EHR) has become a prominent part of outpatient physician practice, with recent studies suggesting that physicians spend over one-half of their workday interacting with the EHR ( Arndt *et al.*, 2017; Young *et al.*, 2018). As a result, the Association of American Medical Colleges lists the ability to document a clinical encounter among its Core Entrustable Professional Activities for Entering Residency, and the American Medical Association has similarly adopted policies encouraging EHR training in medical school and residency. In 2018, the Centers for Medicare and Medicaid Services revised their claims processing manual to allow medical student notes to be used for billing purposes, provided the information is verified and attested to by a supervising physician. With this seismic shift in the role of the medical student, we sought to investigate the educational impact of student EHR use in the ambulatory care setting. Specifically, we aimed to examine 1) how student note-writing is incorporated into clinic workflow, 2) benefits derived from student use of the EHR, and 3) potential areas for improvement in regards to the documentation experience.

## Methods

We conducted a survey of medical students at the Duke University School of Medicine (DuSoM) in Durham, North Carolina (USA) who participated in a longitudinal ambulatory care experience during the 2018-2019 academic year, the first year in which DuSoM implemented billing for student notes. Students at Duke have 3 opportunities to participate in longitudinal ambulatory care experiences: Primary Care Leadership Track (PCLT), Longitudinal Integrated Clerkship (LIC), and Continuity Clinic (CC). PCLT and LIC are ambulatory care-focused clerkship tracks which serve as an alternative to the traditional 2 ^nd^ year clerkship training; students in these programs spend 7-8 months of the year rotating through various primary care and subspecialty clinics. CC is an opportunity for 3 ^rd^ year (research year) medical students to spend one half-day per week with a preceptor of their choice for 8-9 months. In September of 2019, a total of 91 students were emailed an invitation to complete a 19-question survey designed by the authors, which included Likert scale and free-response questions. Survey data was collected and managed using REDCap electronic data capture tools hosted at DuSoM ( Harris *et al.*, 2009). This study was granted exempt status by the DuSoM IRB.

## Results/Analysis

**Figure 1.**
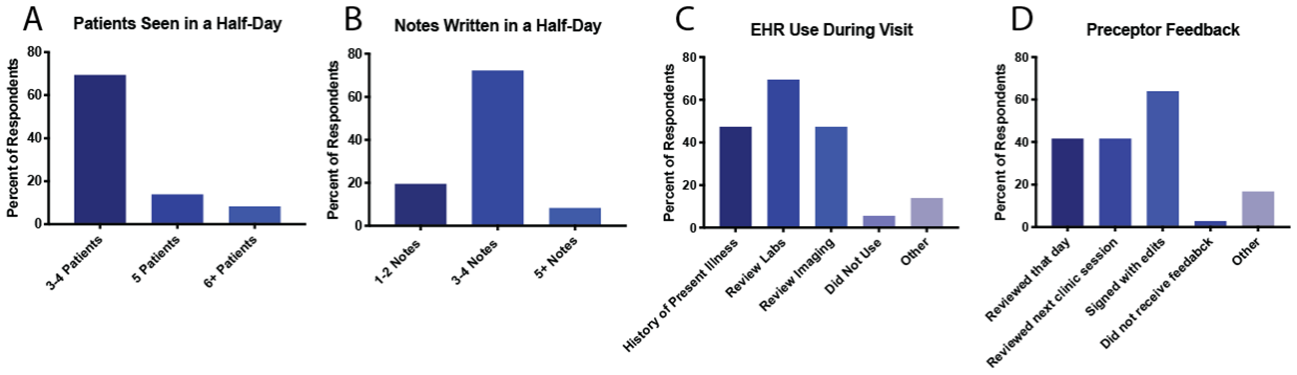
Integration of student documentation into clinic workflow (N = 36). Note for panels C-D, participants could select more than 1 response.

36 students completed the survey (40% response rate). 56% of respondents worked in primary care clinics with the remainder in subspecialty clinics. A majority of students saw 3-4 patients per half-day (69%) and wrote 3-4 notes (72%) ( Figure 1A-B). Almost all (92%) students reported that their notes were used for billing. Major uses of the EHR in the patient room included documenting the HPI (47%) and reviewing pertinent labs (69%) and imaging (47%) ( Figure 1C). Most students received direct feedback from their preceptor on the same day (42%) or during the following clinic session (42%). Many students also received indirect feedback by reviewing preceptor attestations (64%) ( Figure 1D).

**Figure 2.**
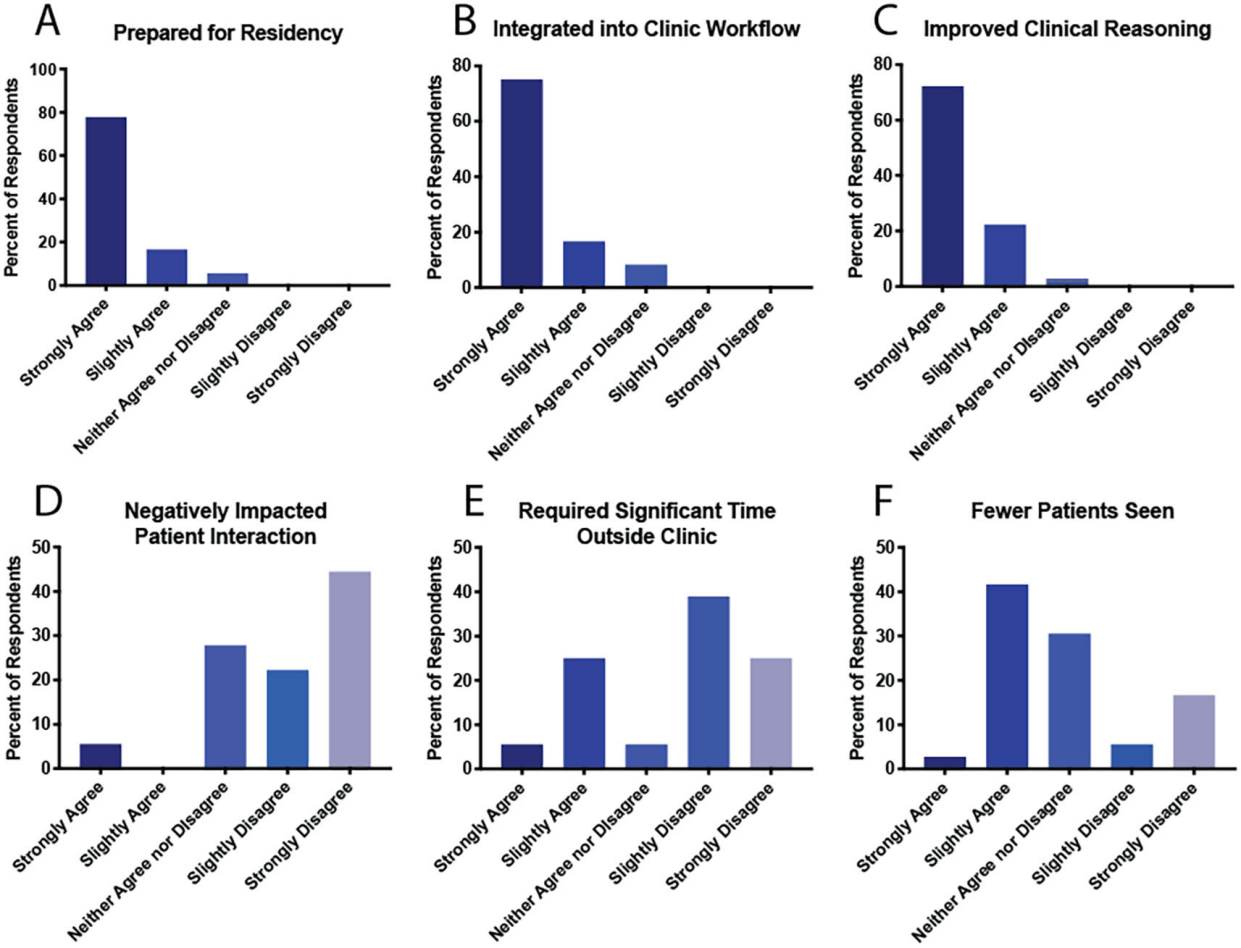
Perceived benefits and disadvantages of medical student documentation (N = 36).

In terms of perceived benefits, nearly all respondents reported feeling more prepared for residency (94% strongly or slightly agree) and more integrated into the clinic workflow (92%) as a result of writing notes ( Figure 2A-B). 94% of students also felt their clinical reasoning skills were improved ( Figure 2C). One student explained, “writing notes in the clinic setting where there are time constraints, I learned to write more quickly and effectively.” Another described how note-writing “helped to consolidate my thinking and assessment of patients in a comprehensive, organized fashion.”

Regarding potential disadvantages, the majority of respondents did not feel that note-writing detracted from patient interactions (67% slightly or strongly disagree), though some felt it reduced the number of patients seen (44% slightly or strongly agree) ( Figure 2D-E). Most students did not feel note-writing to be a significant time burden outside of clinic (64% slightly or strongly disagree) ( Figure 2F). Other difficulties reported included lack of familiarity with billing requirements and preceptor apprehension toward student note-writing.

## Discussion

As medical student notes now become utilized for billing, one potential concern is that the business aspects of note-writing might overshadow the educational benefits. Encouragingly, at our institution, the student response to EHR use in the outpatient setting following these billing changes has been overwhelmingly positive, with a vast majority of students perceiving benefits in regards to residency preparedness, engagement with the clinical team, and clinical reasoning ability. Although we did not investigate the specific aspects of student documentation that correlate with these benefits, it stands to reason that by encouraging students to clearly and concisely articulate patient histories, assessments, and plans, students learn to improve their communication and medical decision-making ability. As preceptors are now mandated to review student notes prior to attesting, the frequency of feedback has also likely increased, further enhancing the learning experience. Finally, because student notes are now being used for billing, as opposed to serving as redundant documentation, students may feel more valued as a member on the medical team.

Reassuringly, and in contrast to older studies ( Rouf, Chumley and Dobbie, 2008), most students did not feel that use of the EHR adversely affected their interactions with patients, which may reflect increasing familiarity with and acceptance of the EHR by students and physicians alike. While some students reported seeing fewer patients as a result of note-writing, the learning derived from each patient may well have been greater. A notable minority of students also reported spending significant amounts of time outside clinic completing notes; as such, preceptors should take care in ensuring that note-writing does not become an undue burden on students. Finally, several students reported a lack of knowledge concerning billing requirements, underscoring a need for additional formalized training; at DuSoM, current training consists of viewing a brief 30-minute video tutorial.

Further study is needed to determine whether preceptors share similarly positive views. A prior nationwide survey of clerkship directors found more neutral opinions toward student EHR use ( Hammoud *et al.*, 2012), with some raising concerns regarding the “copy and paste” nature of notes, which might stifle students’ ability to think for themselves; such practices are known to be widespread among students and physicians ( Heiman *et al.*, 2014). Indeed, in our study, several students reported preceptor apprehension toward student note-writing. Identifying and remedying the specific concerns of these preceptors should be a priority moving forward.

## Conclusion

In this survey of medical students at a major tertiary care center in the United States, we demonstrate that documentation in the EHR remains an important aspect of medical student education following the implementation of billing for medical student notes. Students reported improvements in residency preparedness, clinical reasoning, and integration into the clinical team as a result of note-writing, without adverse effects on patient interaction. Areas for improvement include clarifying billing requirements and overcoming preceptor apprehension toward student note-writing, and future research should focus on how best to optimize training and implementation, with input from all stakeholders.

## Take Home Messages

• Medical student note-writing was well-received by students, with perceived benefits in regards to residency preparedness, integration into the clinical workflow, and clinical reasoning ability.
• Medical students did not feel that note-writing adversely affected patient interactions.
• Areas for improvement include clarifying billing requirements and overcoming preceptor apprehension toward student note-writing.
• Future research should investigate preceptor attitudes toward student note-writing.

## Notes On Contributors

**Charlton Tsai**, **Julia Bellantoni**, and **Omar Martinez-Uribe** are medical students at the Duke University School of Medicine (Durham, NC, USA).

**Bruce Peyser, MD** is a Professor of Medicine in the Division of General Internal Medicine at the Duke University School of Medicine (Durham, NC, USA). He also leads the school’s Primary Care Leadership Track.

## Appendices

### Original Survey Text

This 5-minute survey seeks to examine the experience of medical student clinical documentation in a Longitudinal Integrated Clerkship curriculum. All answers will remain anonymous.Please respond to the survey questions in regards to your experience at the longitudinal clinic where you were most engaged in note writing during the academic year 2018-2019.

1. What type of clinic did you work in?

• Primary care
• Specialty (please specify)

2. How long did you work in the clinic?

• 1-3 months
• 4-6 months
• 7+ months

3. About how many patients did you see during a half-day clinic?

• 1-2 patients per half day
• 3-4 patients per half day
• 4-5 patients per half day
• >5 patients per half day

4. About how many notes did you write during a half-day clinic?

• 1-2 notes per half day
• 3-4 notes per half day
• 4-5 notes per half day
• >5 notes per half day

5. Did the provider utilize your note for billing?

• Yes
• No

6. Did you prechart or preload your notes before clinic?

• Yes
• No

7. How did you use the chart while in the patient room? (select all that apply)

• Write the HPI
• Review lab results with patient
• Review imaging with patient
• Other (please specify)

8. When did you complete your notes?

• While in clinic
• After clinic
• Sometimes in clinic, sometimes after

9. How did you receive feedback on your notes? (select all that apply)

• Preceptor reviewed your note with you that day
• Preceptor reviewed your note during next clinic session
• Preceptor signed note with edits; student reviewed on own time
• I did not receive feedback on notes
• Other (please specify)

10. I felt well prepared/trained to write notes during my longitudinal clinic.

• Strongly agree
• Slightly agree
• Neither agree nor disagree
• Slightly disagree
• Strongly disagree

11. I felt more integrated into the medical team and my clinic’s workflow by writing notes.

• Strongly agree
• Slightly agree
• Neither agree nor disagree
• Slightly disagree
• Strongly disagree

12. Note writing has improved my clinical reasoning skills.

• Strongly agree
• Slightly agree
• Neither agree nor disagree
• Slightly disagree
• Strongly disagree

13. I feel more prepared for residency by writing notes in clinic.

• Strongly agree
• Slightly agree
• Neither agree nor disagree
• Slightly disagree
• Strongly disagree

14. How else has note writing benefited your learning experience? (Free Response)

15. I see fewer patients in clinic when I am expected to write notes.

• Strongly agree
• Slightly agree
• Neither agree nor disagree
• Slightly disagree
• Strongly disagree

16. Note writing requires spending significant periods of time outside of clinic preparing or finishing notes.

• Strongly agree
• Slightly agree
• Neither agree nor disagree
• Slightly disagree
• Strongly disagree

17. Note writing during a clinic visit negatively impacts the way I interact with the patient.

• Strongly agree
• Slightly agree
• Neither agree nor disagree
• Slightly disagree
• Strongly disagree

18. What other disadvantages have you found in note writing? (Free Response)

19. What suggestions do you have for improving the medical student note writing experience? (What has worked well? What could be different?) (Free Response)
