# Supplementary material for: Training in the Era of EHR: Examining the Experience of Medical Student Documentation in the Ambulatory Care Setting
Source: MedEdPublish (2016). 2020 Mar 27;9:56. [Version 1] doi: 10.15694/mep.2020.000056.1 (PMC10697456; doi:10.15694/mep.2020.000056.1)
Supplement: Peer review history

# Reviewer report: Reviewer response for version 1

**DOI:** 10.21956/mep.20022.r30554

**Published:** 2020-05-12

**Authors:** Keith Wilson

**Affiliations:** 1 Dalhousie University

This review has been migrated. The reviewer awarded 4 stars out of 5 The authors present a succinct study on the impact of student EHR use in a clinical setting. The results of this survey showed a positive perception from students. Interestingly, students felt that they were more prepared for residency and that its use expanded their clinical reasoning skills. Additionally, the authors highlighted that students felt more integrated with the team and that this may be driven by their utility (rather than simply providing redundant documentation) – this can be helpful for preceptors to know: that the documenting process can be more than just a required task.It would have been helpful to the reader to have some examples of what students described in the last two free-text questions (disadvantages and suggestions). Although the sample size was small, the conclusions of this study are likely representative of a broader student experience. It might be interesting to take some of what was found to another level: does the use of EHR actually impact clinical reasoning skills? Additionally, exploration of student reasons behind these answers (perhaps using a qualitative lens) would be very helpful in informing future research. The present study will be of interest to any educator or clinician who is using electronic record systems with students.

Reviewer Expertise:

NA

No decision status is available

# Reviewer report: Reviewer response for version 1

**DOI:** 10.21956/mep.20022.r30556

**Published:** 2020-03-30

**Authors:** Deb Halder

This review has been migrated. The reviewer awarded 4 stars out of 5 Note on Title, Abstract and ReferencesThe title is suggestive and it is all inclusive on what it aims to research and who the targets are. The abstract has tended to make an informative summary on the total design, results as well as the directions of study. The references used for the purpose of this research paper is recent, suitable, and symmetrical to the aims and objectives of the paper. Notes on Introduction and MethodThe introductory part of this paper is well-written with the highlight of all necessary tools inserted. Introduction has been able to explain the problem, the objectives of the research, the research questions as well as the hypothesis.But, the method seems to be not in details. It could not explain sampling procedure, data analysis and presentation technique. Without the detail explanation of the procedure, the result cannot be retested by other parties and cannot be proved likewise. Results and DiscussionResults have been presented on ratio scale and the discussion has been on descriptive analytics. The result is details in interpretation though it was not indicated how the findings would be presented.Findings in the discussion part has been proved with reasonable citations. But, the discussion has been found as descriptive rather than analytic and critical evaluation which are the core of discussion. Conclusion and AppendixAn academic research paper should follow third person point of view to retain neutrality and it is more likely adoptable for the construction of conclusion which this paper violates. Conclusion should guide on universal thought or aspect on the researched issues. What was surveyed and found is dealt in previously. Conclusion aims at summing at the general thought with the help of findings and references derived from the paper. The inclusion of the appendix is intelligent task for the authors; it also needs to deal with how it is constructed and validated. There prevail lots of intervening variables in case of emailed generated responses as it cannot ensure who really respond to the questions, what their age limits are etc. The paper has not guided how those limitations were settled.

Reviewer Expertise:

NA

No decision status is available

# Reviewer report: Reviewer response for version 1

**DOI:** 10.21956/mep.20022.r30555

**Published:** 2020-03-28

**Authors:** Trudie Roberts

**Affiliations:** 1 Leeds Institute of Medical Education

This review has been migrated. The reviewer awarded 4 stars out of 5 As EHR become the norm it is important that medical students get experience in this area. So this paper is a useful contribution. It is good to note that students felt this experience would prepare them for residency and not surprising that preceptors were more apprehensive. Interesting that because preceptors had to review the student entries the perhaps unintended (positive) consequence was that the students got more feedback.As a UK physician to understand that one could bill for medical student entries in the EHR was a learning point for me.

Reviewer Expertise:

NA

No decision status is available
